# Clinical and Prognostic Value of Non-Fasting Lipoproteins and Apolipoproteins in Chinese Patients with Coronary Heart Disease

**DOI:** 10.31083/j.rcm2411314

**Published:** 2023-11-09

**Authors:** Junfeng Zhang, Zhengde Tang, Jintong Jiang, Shuying Huang, Huasu Zeng, Jun Gu, Changqian Wang, Huili Zhang

**Affiliations:** ^1^Department of Cardiology, Shanghai Ninth People’s Hospital, Shanghai Jiao Tong University School of Medicine, 200011 Shanghai, China

**Keywords:** lipid, non-fasting state, coronary heart disease, major adverse cardiovascular events, coronary artery stenosis

## Abstract

**Background:**

Lipid profiles differ naturally between individuals and between populations. So far, the data relating to non-fasting lipid profiles has been derived predominantly from studies on Western population. The characteristics and clinical significance of non-fasting lipids in Chinese patients with coronary heart disease (CHD) in response to traditional Chinese diets remain poorly understood.

**Methods:**

A total of 1022 Chinese CHD patients with coronary artery luminal stenosis >40% as diagnosed by coronary artery angiography were enrolled in the study. All patients received standard treatment for CHD, including statins. They were divided into an intermediate stenosis group (luminal stenosis 40–70%, n = 486) or a severe stenosis group (luminal stenosis >70%, n = 536). Their blood lipid profiles were measured in the fasting state, and 4 hours after normal breakfast. All participants were followed up for five years. Major adverse cardiovascular events (MACE) including all-cause death, cardiac death, myocardial infarction, unscheduled coronary revascularization and stroke were recorded.

**Results:**

After normal breakfast intake, patients with intermediate or severe stenosis showed an apparent increase in the levels of triglyceride (TG), remnant cholesterol (RC) and Apo (apolipoprotein) A1 compared to the fasting state, but a significant reduction in the levels of total cholesterol (TC), low-density lipoprotein cholesterol (LDL-C), non-high-density lipoprotein cholesterol (non-HDL-C), Apo B and Apo E. In addition to the traditional risk factors (older age, male, diabetes and smoking) and coronary artery stenosis, the fasting levels of LDL-C and Apo B, as well as non-fasting levels of HDL-C and Apo A1, were identified as independent predictors of 5-year MACE occurrence by multivariate Cox proportional hazards analysis. Patients in the 1st tertile of the non-fasting HDL-C group (<0.86 mmol/L) showed a significantly higher risk of MACE than 3rd tertile (>1.07 mmol/L) (1st tertile: 2.786, 95% CI (confidence intervals) [1.808, 4.293], *p <* 0.001).

**Conclusions:**

This prospective observational study found that lipid profiles in either the fasting or non-fasting states were associated with the long-term risk of MACE in Chinese CHD patients. In addition to the fasting LDL-C level, a low non-fasting HDL-C level may also be an independent risk factors for cardiovascular events. Measurement of lipid profiles during the non-fasting state may be feasible for the management of CHD patients in routine clinical practice in China.

## 1. Introduction

A fasting lipid profile is typically used to assess cardiovascular risk, even though humans are mostly in a non-fasting state during the 24-hour period of each day [1, 2]. Numerous observational studies have in fact demonstrated that normal food intake has minimal effect on lipid and lipoprotein levels [3, 4, 5, 6, 7]. Non-fasting lipid measurement would not only facilitate blood lipid testing by laboratories and clinicians, but also increase patient compliance. Furthermore, various large-scale prospective studies have demonstrated that the association of non-fasting lipid profiles with the risk of cardiovascular diseases is similar to that observed with measurements taken during fasting [3, 4, 8, 9, 10]. A meta-analysis from the Emerging Risk Factors Collaboration have found that non-fasting non-high-density lipoprotein cholesterol (non-HDL-C) and non-fasting calculated low-density lipoprotein cholesterol (LDL-C) were even better for cardiovascular risk prediction than those evaluated in the fasting state [11]. Therefore, non-fasting lipid profiles have been accepted as the clinical standard in Denmark since 2009, based on recommendations from the Danish Society for Clinical Biochemistry [12]. Subsequently, the UK National Institute for Health and Care Excellence (NICE) clinical guideline CG181 endorsed the use of non-fasting lipid profiles for cardiovascular primary prevention [13]. In 2016, the joint consensus statement from the European Atherosclerosis Society (EAS) and the European Federation of Clinical Chemistry also recommended the use of non-fasting blood samples for lipid testing in routine clinical practice [14].

Lipid profiles differ naturally between individuals and between populations. So far, the data relating to non-fasting lipid profiles has been derived predominantly from studies on Western population. The characteristics and clinical significance of non-fasting lipids in Chinese patients with coronary heart disease (CHD) in response to traditional Chinese diets remain poorly understood. Therefore, we conducted a prospective observational study on Chinese CHD patients that examined both their fasting and non-fasting lipid profiles. In addition, we evaluated the predictive value of non-fasting lipid profiles for the risk of cardiovascular events during a 5-year follow-up period.

## 2. Materials and Methods

### 2.1 Study Population

This prospective observational study was carried out at the Shanghai Ninth People’s Hospital, Shanghai JiaoTong University School of Medicine. The study was approved by the hospital ethics review board (2016-256-T191) and conducted in compliance with the declaration of Helsinki. All participants signed a consent form prior to entering the study. A total of 1203 patients with acute or stable chest discomfort and at least one cardiovascular risk factor was screened from January 2015 to April 2017. All participants underwent elective coronary artery angiography after admission. Finally, 1022 patients with intermediate or severe coronary stenosis were enrolled. A total of 181 patients were excluded because they had mild stenosis (<40%) or negligible lesions in the main coronary arteries and branches, or because they had serious lung diseases, severe cardiomyopathy, severe valvular heart disease, severe heart failure, infectious disease, autoimmune disease, familial hyperlipidemia, thyroid disease, severe renal disease, severe liver dysfunction, malignant tumor or some other serious medical illness.

Anti-platelet drugs, statins and/or other lipid-lowering drugs, angiotensin converting enzyme inhibitor (ACEI) or angiotensin II receptor blocker (ARB), β-blocker or nitrates were routinely used in all patients. Patients underwent coronary artery bypass graft surgery or percutaneous coronary intervention when specialists deemed the procedure was necessary and beneficial. All laboratory and clinical information as well as demographic data were collected.

### 2.2 Coronary Artery Angiography 

Elective coronary angiography (Judkin’s technique) was performed on all participants after admission. Two independent interventional experts who were blind to the clinical information separately quantified the severity of stenosis in the coronary artery. Luminal stenosis with a diameter narrowing of >70% in any of the main coronary arteries was defined as a severe lesion. These included the right coronary artery (RCA), left main artery (LM), left anterior descending artery (LAD), left circumflex coronary artery (LCX), as well as their main branches (vessel diameter ≥2.5 mm). Luminal stenosis of 40–70% in any of the main coronary arteries or main branches was defined as an intermediate lesion.

### 2.3 Blood Collection and Laboratory Assays 

Blood samples were collected after a 12-hour overnight fast and 4 hours after a daily breakfast according to the participant’s dietary habit. Plasma levels of triglycerides (TG), total cholesterol (TC), high-density lipoprotein cholesterol (HDL-C) and low-density lipoprotein cholesterol (LDL-C) were assayed by an automated biochemistry analyzer (Siemens Advia 2400, Siemens Healthcare Diagnostics Inc., Deerfield, IL, USA). LDL-C was calculated using the Friedewald equation [LDL-C = TC – (HDL-C) – (TG / 2.2)] when TG was <4.5 mmol/L. A higher TG levels, LDL-C was measured directly. Remnant cholesterol (RC) and non-HDL-C levels were calculated by the following equations: [RC = TC – (HDL-C) – (LDL-C)] and [non-HDL-C = TC – (HDL-C)].

### 2.4 Follow-Up and Major Adverse Cardiovascular Events

All patients visited their doctors every three months in our outpatient clinic. Patients were interviewed by telephone if they could not attend their scheduled clinic appointment. They were followed up for 5 years and any major adverse cardiovascular events (MACE) during this time were recorded. MACE was defined as the composite of all cause death, cardiac death, myocardial infarction, unscheduled coronary revascularization, and stroke. Patient follow-up was 100% complete.

### 2.5 Statistical Analysis 

IBM SPSS Statistics 23.0 (IBM Corp., Armonk, NY, USA) software was used to perform statical analysis. The distribution of lipid profiles was confirmed by the Shapiro-Wilk and Kolmogorov-Smirnov tests. Some lipid parameters in the fasting and non-fasting states were not normally distributed, hence mostly non-parametric statistical analysis was used in this study. Categorical variables were expressed as a percentage, while continuous variables were presented as the median [first quartile, third quartile]. Mann-Whitney U test was used to compare continuous variables between two independent groups. The chi-square test was used to compare categorical variables. Differences between fasting and non-fasting lipid profiles within a single group was compared by the Wilcoxon signed-rank test. Multivariate logistic regression analysis (forward conditional) was performed to identify independent factors associated with the severity of coronary artery stenosis. Lipid profiles and clinical characteristics with a *p* value < 0.05 in univariate analysis were included in multivariate logistic regression analysis. Odds ratios (OR) and 95% confidence intervals (CI) were calculated. Hazard ratios (HR) for the risk of MACE during the 5-year follow-up period were estimated using multivariate Cox proportional hazards regression analysis (forward conditional). Lipid profiles and clinical characteristics with a *p* value < 0.05 in univariate analysis were included in multivariate Cox regression analysis. Correlations between TC, HDL-C, LDL-C, non-HDL-C, Apo (apolipoprotein) A1, and Apo B were assessed by Spearman correlation analysis. Statistical significance was considered when the *p *value was <0.05.

## 3. Results

### 3.1 Demographic and Clinical Characteristics

Table 1 shows the baseline characteristics of the study participants. A total of 1022 CHD patients with angiographically-determined coronary artery stenosis of >40% were included in this study. Of these, 486 patients had intermediate coronary artery luminal stenosis (40–70%) and 536 had severe luminal stenosis (>70%). Compared with the intermediate stenosis group, patients in the severe stenosis group were older, more likely to be men, more likely to smoke, more likely to have a prior CHD history, as well as having a higher incidence of hypertension and diabetes mellitus. Patients with severe stenosis also tended to have a higher incidence of acute coronary syndrome (ACS) and revascularization, as well as higher levels of troponin I, B-type natriuretic peptide (BNP) and C-reaction protein (CRP) compared to patients with intermediate stenosis. As expected, patients with severe coronary artery stenosis therefore tended to have more cardiovascular risk factors and complications. The majority of participants received statins for at least three months prior to enrolling in this study. Statins were administered to 356 and 446 patients in the intermediate and severe stenosis groups, respectively (73.2% *vs*. 83.2%, *p* < 0.001).

**Table 1.** **Demographic characteristics and baseline clinical features of Chinese CHD patients with intermediate or severe coronary artery stenosis**.

		Intermediate stenosis (n = 486)	Severe stenosis (n = 536)	*p* value
Age (years)		66 [60, 73]	68 [61, 75]	0.006
Male		252 (51.9%)	374 (69.8%)	<0.001
BMI (kg/$m^{2}$)		24.49 [22.27, 26.57]	24.75 [22.49, 26.81]	0.316
Type of CHD				
	ACS	33 (6.8%)	134 (25.0%)	<0.001
	Non-ACS	453 (93.2%)	402 (75.0%)	<0.001
Revascularization				
	PCI	0 (0%)	420 (78.4%)	<0.001
	CABG	0 (0%)	11 (2.1%)	<0.001
	${\mathrm{Other}}^{1}$	486 (100%)	105 (19.6%)	<0.001
Medical history				
	${\mathrm{Smoker}}^{2}$	165 (34.0%)	260 (48.5%)	<0.001
	Hypertension	323 (66.5%)	381 (71.1%)	0.120
	Diabetes mellitus	108 (22.2%)	202 (37.7%)	<0.001
	Prior history of ${\mathrm{CHD}}^{3}$	114 (23.5%)	290 (54.1%)	<0.001
	History of atrial fibrillation	61 (12.6%)	72 (14.6%)	0.710
	COPD	32 (6.6%)	40 (7.5%)	0.626
	Family history of CVD	260 (53.5%)	277 (51.7%)	0.573
Medications				
	Aspirin and/or thienopyridine	465 (95.7%)	509 (96.8%)	0.408
	Anticoagulants	60 (12.3%)	72 (14.6%)	0.641
	Beta blocker	115 (23.6%)	133 (24.9%)	0.715
	ACEI/ARB	129 (26.5%)	153 (28.7%)	0.484
	CCB	68 (14.0%)	65 (12.1%)	0.403
	Statins	356 (73.2%)	446 (83.2%)	<0.001
	Other lipid-lowering ${\mathrm{drugs}}^{4}$	28 (5.8%)	45 (9.1%)	0.114
Laboratory variables				
	Troponin I (ng/mL)	0.00 [0.00, 0.01]	0.01 [0.00, 0.05]	<0.001
	BNP (pg/mL)	49 [24.75, 98.50]	66 [33.00, 182.00]	<0.001
	CRP (mg/L)	1.28 [1.28, 4.29]	1.81 [1.28, 6.50]	<0.001
	Creatine level (µmol/L)	78.00 [67.00, 93.00]	81.00 [67.00, 97.00]	0.118

Values are expressed as percentage or median [first quartile, third quartile]. ACS, acute coronary syndrome; ACEI, angiotensin converting enzyme inhibitor; ARB, angiotensin II receptor blocker; BNP, B-type natriuretic peptide; CABG, coronary artery bypass graft; CHD, coronary heart disease; COPD, chronic obstructive pulmonary disease; CRP, C-reaction protein; CVD, cardiovascular disease; PCI, percutaneous coronary intervention; BMI, body mass index; CCB, calcium channel blocker. ^1^ Other refers to those who did not meet the standard for revascularization or refused to receive revascularization. ^2^ Smokers include current smokers or former smokers who have quitted cigarette smoking for less than 10 years. ^3^ Prior history of CHD include prior myocardial infarction, prior coronary artery revascularization and documented coronary artery stenosis by angiography. ^4^ Other lipid-lowering drugs include cholesterol absorption inhibitor, fibrates, fish oil etc.

### 3.2 Fasting and Non-Fasting Lipid Profiles

Lipoproteins are spherical particles that have a central core containing cholesterol esters and triglycerides, surrounded by free cholesterol, phospholipids, and apolipoproteins. They are divided mainly into five types, chylomicrons, very low-density lipoprotein (VLDL), intermediary density lipoprotein (IDL), low-density lipoprotein (LDL), and high-density lipoprotein (HDL), based on their relative size and densities. Different types of lipoproteins contain different apolipoproteins, which facilitate their function in cholesterol transportation and lipid metabolism. Table 2 shows the fasting and non-fasting lipid profiles in the study population. Four hours after normal breakfast intake, patients with intermediate or severe stenosis experienced an apparent increase in the levels of TG, RC and Apo A1 compared to the fasting state, but a significant decrease in the levels of TC, LDL-C, non-HDL-C, Apo B and Apo E (Table 2). Compared to the fasting state, the non-fasting level of HDL-C decreased significantly in the severe stenosis group but not in the intermediate stenosis group. After adjusted the baseline characteristics (age, gender, smoking and diabetes), the alterations of lipid profiles from fasting to non-fasting states in both groups remained almost unchanged (**Supplementary Table 1**).

**Table 2.** **Fasting and non-fasting lipid profiles in Chinese CHD patients with intermediate or severe coronary artery stenosis**.

	Intermediate stenosis (n = 486)			Severe stenosis (n = 536)		
	Fasting	Non-fasting	*p* ${\mathrm{value}}^{1}$	Fasting	Non-fasting	*p* ${\mathrm{value}}^{2}$
TC (mmol/L)	4.18 [3.61, 4.86]	4.08 [3.52, 4.69]	0.001	3.93 [3.23, 4.64]	3.68 [3.12, 4.33]	<0.001
TG (mmol/L)	1.45 [1.07, 2.02]	1.63 [1.19, 2.25]	<0.001	1.52 [1.12, 2.14]	1.65 [1.20, 2.31]	<0.001
LDL-C (mmol/L)	2.36 [1.87, 2.94]	2.21 [1.72, 2.72]	<0.001	2.16 [1.62, 2.81]	1.89 [1.45, 2.46]	<0.001
HDL-C (mmol/L)	1.02 [0.87, 1.23]	1.01 [0.87, 1.22]	0.703	0.92 [0.77, 1.11]	0.90 [0.77, 1.08]	<0.001
RC (mmol/L)	0.66 [0.49, 0.91]	0.74 [0.54, 1.02]	<0.001	0.69 [0.50, 0.97]	0.75 [0.55, 1.05]	<0.001
Non-HDL-C (mmol/L)	3.14 [2.55, 3.73]	3.00 [2.48, 3.61]	<0.001	2.97 [2.31, 3.66]	2.72 [2.21, 3.32]	<0.001
Apo A1 (g/L)	1.11 [0.99, 1.25]	1.17 [1.04, 1.30]	<0.001	1.05 [0.93, 1.18]	1.08 [0.97, 1.22]	<0.001
Apo B (g/L)	0.83 [0.69, 1.00]	0.80 [0.68, 0.96]	<0.001	0.82 [0.65, 1.00]	0.75 [0.63, 0.91]	<0.001
Apo E (mg/dL)	4.13 [3.38, 5.03]	4.02 [3.40, 4.96]	0.008	3.85 [3.11, 4.86]	3.69 [2.97, 4.64]	<0.001

Values are expressed as median [first quartile, third quartile]. CHD, coronary heart disease; Apo, apolipoprotein; HDL-C, high density lipoprotein cholesterol; LDL-C, low density lipoprotein cholesterol; RC, remnant cholesterol; TC, total cholesterol; TG, triglyceride. ^1^ Comparison of lipid profiles between fasting state and non-fasting state in intermediate stenosis group. ^2^ Comparison of lipid profiles between fasting state and non-fasting state in severe stenosis group.

### 3.3 Fasting and Non-Fasting Lipid Profiles, and the Severity of Coronary Artery Stenosis

Univariate logistic regression analysis was performed to identify potential factors associated with the severity of coronary artery stenosis. Various clinical features (age, male, smoker, diabetes, prior history of CHD), fasting and non-fasting levels of TC, lipoproteins (LDL-C and HDL-C) and apolipoproteins (Apo A1, Apo B and Apo E) were significantly associated with the severity of coronary artery stenosis (**Supplementary Tables 2,3**). The levels of Apo B and non-HDL-C were highly correlated with LDL-C, while HDL-C was strongly associated with Apo A1 (**Supplementary Fig. 1**). Therefore, lipoproteins and apolipoprotein levels were entered separately into multivariate logistic regression models. The non-HDL-C level was not included in multivariate regression analysis. In the fasting state, multivariate logistic regression model 1 and 3 found that HDL-C (OR 0.262, 95% CI 0.158–0.436, *p* < 0.001) and Apo A1 (OR 0.198, 95% CI 0.099–0.396, *p* < 0.001) were negatively associated with the stenosis severity. A similar pattern was seen in the multivariate regression model 2 and 4 for the non-fasting state, with HDL-C (OR 0.177, 95% CI 0.104–0.303, *p* < 0.001), Apo A1 (OR 0.158, 95% CI 0.077–0.324, *p* < 0.001) and Apo E (OR 0.880, 95% CI 0.801–0.967, *p* < 0.001) being significantly associated with the severity of luminal stenosis. Multivariate regression models also found that male, diabetes mellitus and a prior history of CHD were associated with an increased risk of severe luminal stenosis.

### 3.4 Fasting and Non-Fasting Lipid Profiles and Clinical Outcomes during 5-Year Follow-Up 

During the 5-year follow-up period, 43 patients (8.85%) in the intermediate stenosis group and 113 patients (21.08%) in the severe stenosis group experienced a MACE. Multivariate Cox regression models that were adjusted for non-lipid classical risk factors (age, male, smoking, diabetes and stenosis severity) found that fasting levels of lipoproteins (LDL-C and HDL-C) and apolipoproteins (Apo A1 and Apo B) were strongly associated with the risk of MACE (**Supplementary Tables 4,5**). Multivariate Cox regression models also showed that non-fasting levels of LDL-C, HDL-C, Apo A1 and Apo B had similar associations with MACE (**Supplementary Tables 4,5**). LDL-C and Apo B in either the fasting (LDL-C: HR 1.592, 95% CI 1.349–1.878; Apo B: HR 6.538, 95% CI 3.614–11.827; both *p* < 0.001) or non-fasting (LDL-C: HR 1.657, 95% CI 1.381–1.987; Apo B: HR 5.350, 95% CI 2.793–10.249; both *p* < 0.001) states were both associated with an increased risk of MACE. In contrast, HDL-C and Apo A1 in the fasting (HDL-C: HR 0.248, 95% CI 0.122–0.504; Apo A1: HR 0.199, 95% CI 0.083–0.477; both *p* < 0.001) and non-fasting (HDL-C: HR 0.130, 95% CI 0.060–0.280; Apo A1: HR 0.128, 95% CI 0.054–0.305; both *p* < 0.001) states were negatively correlated with the risk of MACE. However, univariate Cox regression analysis showed that TC and TG levels in both the fasting and non-fasting states were not associated with the risk of MACE during the 5-year follow-up period (**Supplementary Tables 4,5**).

Both non-fasting and fasting levels of lipoproteins (LDL-C and HDL-C) and apolipoproteins (Apo A1 and Apo B) were included in multivariate Cox regression analysis in order to compare their prognostic value. As shown in Table 3, fasting LDL-C and Apo B as well as non-fasting HDL-C and Apo A1 were found to be independent predictors for MACE during the 5-year follow-up. This was in addition to the traditional risk factors of older age, diabetes, smoking and stenosis severity of coronary artery.

**Table 3.** **Fasting and non-fasting lipid profiles and the risk for 5-year MACE occurrence**.

		Multivariate Cox regression		
		HR	95% CI	*p* value
Model 1 (Lipoproteins)				
	Coronary artery stenosis severity	1.646	1.137–2.383	0.008
	Age	1.023	1.007–1.040	0.005
	${\mathrm{Smoker}}^{1}$	1.455	1.054–2008	0.023
	Diabetes mellitus	1.852	1.343–2.554	<0.001
	Fasting LDL-C (mmol/L)	1.628	1.380–1.921	<0.001
	Non-fasting HDL-C (mmol/L)	0.138	0.064–0.296	<0.001
Model 2 (Apolipoproteins)				
	Coronary artery stenosis severity	1.777	1.232–2.563	0.002
	Age	1.024	1.008–1.041	0.003
	Diabetes mellitus	1.838	1.333–2.532	<0.001
	Fasting Apo B (g/L)	6.038	3.352–10876	<0.001
	Non-fasting Apo A1 (g/L)	0.111	0.045–0.274	<0.001

Values are expressed as hazard ratio (HR) and 95% confidence intervals (CI). Apo, apolipoprotein; HDL-C, high density lipoprotein cholesterol; LDL-C, low density lipoprotein cholesterol; MACE, major adverse cardiovascular events. ^1^Smokers include current smokers or former smokers who have quitted cigarette smoking for less than 10 years.

### 3.5 Non-Fasting HDL-C and Clinical Outcome during a 5-Year Follow-Up

The significance of non-fasting HDL-C was then investigated in this study, because the clinical relevance of fasting LDL-C and Apo B in CHD is already well-established. The entire cohort was divided into three groups according to tertiles of non-fasting HDL-C. The baseline clinical characteristics and laboratory test results for the three groups are shown in **Supplementary Table 6**. A total of 353 (34.5%), 335 (32.8%), and 334 (32.7%) patients were categorized into 1st tertile (non-fasting HDL-C <0.86 mmol/L), 2nd tertile (non-fasting HDL-C 0.86–1.07 mmol/L), and 3rd tertile (non-fasting HDL-C >1.07 mmol/L) groups, respectively. Patients in the 1st tertile group tended to be younger, male and have a prior history of CHD, as well as having higher BMI (body mass index), statin use, troponin I and creatinine levels than the other groups. Patients in the 1st tertile group also had a significantly higher percentage of severe coronary artery stenosis and a lower percentage of intermediate stenosis than those in the 2nd and 3rd tertile groups.

**Fig. 1.**
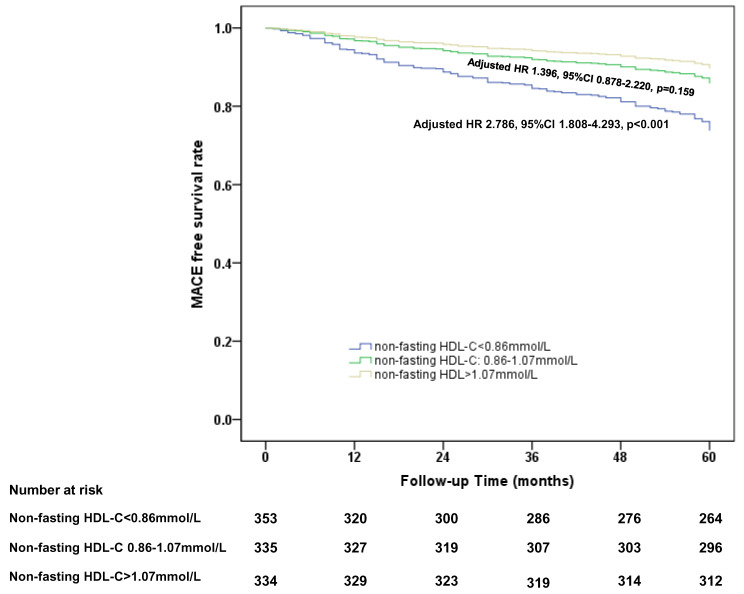
**Cox proportional hazards regression analysis of 5-year MACE free survival rate in CHD patients divided by the tertiles of non-fasting HDL-C level**. HDL-C, high density lipoprotein cholesterol; HDL, high-density lipoprotein; CHD, coronary heart disease; MACE, major adverse cardiovascular events; HR, hazard ratio; CI, confidence intervals.

During the 5-year follow-up period, 89 (25.2%), 39 (11.6%) and 22 (6.6%) MACE were recorded in the 1st, 2nd and 3rd tertile groups, respectively. The MACE-free survival rate in the 1st tertile group was significantly lower than that observed in the 3rd tertile group (Fig. 1). After adjustment for baseline clinical characteristics (age, gender, BMI and fasting lipids), patients in the 1st tertile group showed a significant higher risk of MACE during the 5-year follow-up period compared to that in the 3rd tertile group (1st tertile: 2.786, 95% CI [1.808, 4.293], *p* < 0.001).

Stratified analysis of the association between non-fasting HDL-C and MACE was conducted according to the baseline clinical characteristics. As shown in Table 4, the association between non-fasting HDL-C and MACE was independent of male, older age, BMI, stenosis severity and a prior history of CHD. The cutoff value of non-fasting HDL-C level associated with MACE-free survival during the 5-year follow-up period was >0.942 mmol/L with 54.8% sensitivity and 66.7% specificity [area under curve (AUC): 0.627, 95% CI 0.579–0.674, *p* < 0.01] (**Supplementary Fig. 2**).

**Table 4.** **Stratified analysis of the association between non-fasting HDL-C and MACE by potential risk factors**.

Subgroup		n	3rd Tertile	1st Tertile		2nd Tertile		*p* value for interaction
				Adjusted HR (95% CI)	*p* value	Adjusted HR (95% CI)	*p* value	
Age								0.058
	<65 years	450	Ref	2.487 (1.356–4.563)	0.003	0.680 (0.302–1.531)	0.352	
	≥65 year	572	Ref	2.504 (1.435–4.370)	0.001	1.887 (1.063–3.352)	0.030	
Gender								0.076
	Male	626	Ref	2.369 (1.455–3.859)	0.001	1.078 (0.614–1.894)	0.793	
	Female	396	Ref	2.432 (1.138–5.198)	0.022	1.889 (0.857–4.162)	0.115	
BMI		1022	Ref	2.609 (1.730–3.933)	<0.001	1.387 (0.876–2.194)	0.163	0.690
Stenosis								0.067
	Intermediate	486	Ref	3.249 (1.500–7.040)	0.003	1.700 (0.763–3.785)	0.194	
	Severe	536	Ref	1.757 (1.081–2.855)	0.023	1.055 (0.603–1.843)	0.852	
Prior history of CHD								0.919
	Yes	404	Ref	2.535 (1.356–4.739)	0.004	1.237 (0.595–2.537)	0.568	
	No	616	Ref	2.407 (1.397–4.149)	0.002	1.341 (0.742–2.423)	0.331	

HDL-C, high density lipoprotein cholesterol; CHD, coronary heart disease; MACE, major adverse cardiovascular events; HR, hazard ratio; CI, confidence intervals; BMI, body mass index.

## 4. Discussion

This prospective observational study investigated the clinical significance of fasting and non-fasting lipid profiles in Chinese CHD patients. All participants had >40% luminal stenosis in the main branches of coronary arteries and most had received statins for at least three months before enrolment in the study. Compared to the fasting state, CHD patients with intermediate (40–70%) or severe stenosis (>70%) showed changes in the levels of lipoproteins (LDL-C and HDL-C) and apolipoproteins (Apo A1, Apo B and Apo E) four hours after normal food intake. Although the lipid profiles changed in response to the daily diet, both fasting and non-fasting levels of lipoproteins and apolipoproteins showed similar predictive value for MACE in Chinese CHD patients. This was observed, regardless of the severity of coronary artery stenosis, age, gender, smoking status or diabetes. LDL-C and Apo B levels in a fasting state showed strong associations with an increased risk of MACE in CHD (HR 1.628 and 6.038, respectively; both *p* < 0.001). In the contrary, non-fasting levels of HDL-C and Apo A1 were negatively associated with the risk of MACE (HR 0.138 and 0.111, respectively; both *p* < 0.001). Although the use of fasting lipid measurements is currently recommended by the guidelines in China, our findings suggest that non-fasting lipid levels could also be used for CHD management in routine clinical practice.

For many years, most guidelines or statements for the assessment of cardiovascular risk have recommended measurement of lipid profiles in a fasting state. This may be due to the dynamic changes observed in some lipid components, especially triglyceride during a postprandial test (high-fat tolerance). In fact, people eat much less fat in daily life and are mostly in a non-fasting state during 24-hour period of each day. Several large-scale, population-based studies that included men, women, children and diabetic patients have compared the fasting and non-fasting lipid levels in response to daily food intake [3, 4, 5, 6, 7]. These found a slight increase in non-fasting TG levels (0.1–0.3 mmol/L, or 10–21% increase from the fasting state) [3, 4, 5, 6, 7], and small decreases in non-fasting TC levels (0.1–0.3 mmol/L, or a 1–8% reduction from the fasting state) and LDL-C (0.1–0.3 mmol/L, or 4–9% reduction from the fasting state) [3, 4, 6, 7]. However, the changes observed between fasting and non-fasting HDL-C levels were inconsistent. Based on the Copenhagen General Population Study, the maximum mean changes at 1–6 h after habitual meals were –0.1 mmol/L for HDL-C [3]. Some studies showed the non-fasting HDL-C level remained unchanged in children aged 12 years or older, as well as in a large community-based cohort [6, 7]. Taken together, these studies suggest that lipids and lipoproteins changed only slightly in response to normal food intake in men, women and children.

The effect of daily food intake on plasma lipids has been extensively investigated in Western-population-based studies. However, the changes in non-fasting lipids in the Chinese CHD population following the intake of traditional Chinese food intake have rarely been investigated. Previous studies showed the overall levels of TC and LDL-C gradually decreased from 1 to 4 hours following normal food intake compared with fasting levels, while TG levels increased for up to 6 hours after the last meal [3, 15]. Therefore, the non-fasting blood samples used for lipid measurement in the present study were collected 4 hours after breakfast. CHD patients with intermediate or severe stenosis showed significant reductions in the levels of non-fasting TC, LDL-C and non-HDL-C compared to the fasting state. An obvious increase in the non-fasting levels of TG and RC was observed only in CHD patients with intermediate stenosis, whereas a significant reduction in non-fasting HDL-C was observed in the severe, but not intermediate stenosis group. Thus, in the present study the overall changes in lipoprotein levels observed between the fasting and non-fasting states are in line with those reported in previous large-scale, cohort studies of Western populations [3, 4, 5, 6, 7]. Alterations in lipoproteins after food intake may be attributed to overproduction and decreased catabolism of triglyceride-rich lipoproteins and their remnants, especially in patients with hypertriglyceridemia, metabolism syndrome or diabetes [16, 17]. Transfer of triglycerides from triglyceride-rich lipoproteins to HDL and LDL particles in exchange for cholesteryl esters leads to reduced HDL-C and LDL-C levels in the non-fasting state [18].

Of note, the present study found that significant changes in apolipoprotein levels between fasting and non-fasting states were observed in CHD patients with either intermediate or severe stenosis. However, the Danish general population study, the Copenhagen General Population Study, and the Copenhagen City Heart Study showed that Apo B and Apo A1 do not change in response to normal food intake [3]. This discrepancy with the current findings may be due to different study populations and dietary habits.

The current study also evaluated the significance of non-fasting lipoproteins and apolipoproteins in terms of the long-term risk of MACE in CHD patients. Both the fasting and non-fasting levels of lipoproteins (LDL-C and HDL-C) and apolipoproteins (Apo A1 and Apo B) were identified by multivariate Cox proportional hazards analysis as being independent predictors of MACE during 5-year follow-up. To compare their prognostic value, non-fasting and fasting levels of LDL-C, HDL-C, Apo A1 and Apo B were simultaneously included in multivariate Cox regression models. Only fasting LDL-C and Apo B as well as non-fasting HDL-C and Apo A1 remained to be independent predictors for MACE risk. This was in addition to the traditional risk factors (older age, diabetes, smoking) and the stenosis severity of coronary artery. Our findings are consistent with those of several large-scale prospective studies with long-term follow-up that found non-fasting lipid levels were equally robust as predictors of cardiovascular risk and mortality as fasting lipid profiles [3, 4, 8, 9, 10]. A meta-analysis from the Emerging Risk Factors Collaboration assessed 68 long-term prospective studies involving >300,000 individuals (mostly Europe and North America) for correlations between major lipid and apolipoprotein levels and the risk of vascular disease [11]. The strength of the association between lipoproteins and CHD risk was not attenuated in the 20 studies that used non-fasting lipid measurements, with the HR for vascular disease risk and lipid levels being at least as strong in the non-fasting state as in the fasting state [11].

In addition to lipoproteins, the present study investigated the predictive value of fasting and non-fasting apolipoprotein levels for MACE risk. Fasting Apo B and non-fasting Apo A1 were found to provide additive information for the prediction of MACE risk during a 5-year follow-up in Chinese CHD patients. The AMORIS (the apolipoprotein mortality risk) study similarly analyzed Apo A1 and Apo B as predictors of cardiac risk in large healthy populations. Apo B, Apo A1 and the Apo B/Apo A1 ratio were found to provide additional information for predicting the risk of fatal myocardial infarction to that of LDL-C alone [19]. The case-control INTERHEART (a large case-control study of acute myocardial infarction in 52 countries and sponsored by the World Health Organization) study found that the non-fasting Apo B/Apo A1 ratio was better than all other lipid parameters for predicting the risk of acute myocardial infarction in different ethnic, gender, and age groups, and therefore proposed its use in worldwide clinical practice [20]. Taken together, these findings indicate that non-fasting apolipoprotein levels may be quite valuable for cardiovascular risk management, and thereby warranting further investigation.

Apo A5 is important in TG metabolism because it activates lipoprotein lipase (LPL)-mediated triacylglycerol lipolysis [21, 22]. Various single nucleotide polymorphisms (SNPs) in Apo A5 have been identified in Chinese population, including for example rs2075291 c.553G>T, G185C [23, 24]. Animal and population studies have demonstrated that SNPs in *Apo A5* contribute to the susceptibility for CHD in the Chinese population [21, 22, 23, 24]. However, the present study found that fasting and non-fasting levels of TG were not predictors for MACE during 5-year follow-up of Chinese CHD patients, possibly because patients with familial hypertriglyceridemia were excluded. Also, Chinese breakfast is not high in fats. Four hours after breakfast, the non-fasting TG levels did not increase as greatly as observed in Western countries.

To further investigate the clinical significance of non-fasting HDL-C in CHD patients, we divided the study population into three groups according to tertiles of the non-fasting HDL-C level. After 5-year follow-up, the risk of MACE in CHD patients with lower levels of non-fasting HDL-C was approximately 2.5 times higher than that of patients with the highest tertile (1st tertile group, adjusted HR: 2.786). The predictive value of non-fasting HDL-C for MACE was independent of the severity of coronary artery stenosis and of other conventional risk factors. Our findings shed new light on the significance of non-fasting HDL-C as a predictor of MACE in Chinese CHD patients with statin therapy. However, whether a quantitative elevation of plasma HDL-C is beneficial for cardiovascular disease prevention continues to be debatable. Concomitant diseases, drugs, dietary habit and exercise also influence the serum levels of HDL-C. HDL has several structural or functional properties, including reverse cholesterol transport, anti-inflammation, antioxidant effect, or inhibition of platelet aggregation [25]. Functional HDL is likely to be as important as HDL-C level in reducing CHD risk. Some patients with atherosclerotic cardiovascular disease (ASCVD) may have normal or even high HDL-C level but dysfunctional HDL [26]. Therefore, more research is indispensable to evaluate the association of HDL functionality with cardiovascular risk. Potential new treatment based on HDL function may improve the clinical outcome in CHD when added to statin therapy.

In 2009, the Danish Society of Clinical Biochemistry made an official recommendation on the use of lipid measurements in the non-fasting state for cardiovascular risk prediction [12]. Subsequently, the American Heart Association (AHA) [27], the National Institute for Health and Care Excellence (NICE) [13], the European Atherosclerosis Society and the European Federation of Clinical Chemistry [28], the Canadian Cardiovascular Society [29, 30] and other societies [31] updated their guidelines to recommend the use of non-fasting lipid profiles for cardiovascular risk prediction. Moreover, some major statin trials have also used non-fasting blood samples for lipid assessment, including the Heart Protection Study [32], the Anglo-Scandinavian Cardiac Outcomes Trial [33], and the Study of the Effectiveness of Additional Reductions in Cholesterol and Homocysteine [34]. Although prospective studies have demonstrated the advantages and clinical significance of non-fasting lipids for cardiovascular risk prediction, studies on its cost-effectiveness are still lacking [35, 36, 37]. Driver *et al*. [38] noted that clinicians should carefully consider the clinical scenarios (initial cardiovascular risk assessment, residual risk of CHD, diagnosis of familial hyperlipidemia or metabolic syndrome, etc.) when choosing between the use of fasting and non-fasting lipids.

Currently in China, lipid levels in the fasting state are still used routinely for cardiovascular risk assessment. Our study has provided preliminary evidence of the value of non-fasting lipoproteins and apolipoproteins for cardiovascular risk assessment of Chinese CHD patients. Lin *et al*. [15] have also suggested the non-fasting LDL-C level could be used to guide the treatment of Chinese CHD patients if the fasting LDL-C level is <1.4 mmol/L. In summary, more research on large Chinese population cohorts is essential to fully evaluate the association between non-fasting lipid profiles and cardiovascular risk. If non-fasting lipid profiles are accepted worldwide for the assessment of cardiovascular risk, this would greatly simplify clinical care for medical practitioners and patients.

This study has some limitations that should be acknowledged. Firstly, we investigated CHD patients with intermediate or severe stenosis, most of whom received statin therapy. Therefore, our findings cannot be applied to newly diagnosed CHD patients who have yet to receive standard treatment. Secondly, the lipid profiles were not regularly monitored and hence the percentage of patients who reached the target LDL-C level (<1.4 mmol/L) was not carefully evaluated during follow-up. Thirdly, non-fasting lipid profiles are affected by food habits, but we did not examine the patients’ dietary intake, alcohol intake or the use of supplements in this study.

## 5. Conclusions

The present study demonstrated that in addition to classical risk factors and the severity of coronary artery stenosis, the lipoprotein and apolipoprotein levels in both fasting and non-fasting states were independent predictors of the long-term risk of MACE in Chinese CHD patients. Measurement of the lipid profile in the non-fasting state may therefore be a rational and feasible approach for the management of cardiovascular risk in Chinese CHD patients. Non-fasting HDL-C level may provide additional information for CHD risk management in routine clinical practice in China, in addition to fasting LDL-C.

## Data Availability

The datasets used and analyzed during the current study are available from the corresponding author on reasonable request.

## Supplementary Material

Supplementary material associated with this article can be found, in the online version, at https://doi.org/10.31083/j.rcm2411314.
