# Supplementary material for: Clinical and Prognostic Value of Non-Fasting Lipoproteins and Apolipoproteins in Chinese Patients with Coronary Heart Disease
Source: Rev Cardiovasc Med. 2023 Nov 9;24(11):314. doi: 10.31083/j.rcm2411314 (PMC11272848; doi:10.31083/j.rcm2411314)
Supplement: Supplementary file 1 [file 2153-8174-24-11-314-s1.docx]

**Supplementary Materials**

**Table 1. Adjusted comparisons between fasting and non-fasting lipid profiles in Chinese CHD patients with intermediate or severe coronary artery stenosis.**

|  | | *p* value (Fasting vs. non-fasting lipoproteins) | | | | | | *p* value (Fasting vs. non-fasting apolipoproteins) | | |
| --- | --- | --- | --- | --- | --- | --- | --- | --- | --- | --- |
|  | | TC  (mmol/L) | TG  (mmol/L) | LDL-C  (mmol/L) | HDL-C  (mmol/L) | RC  (mmol/L) | Non-HDL-C  (mmol/L) | Apo A1  (g/L) | Apo B  (g/L) | Apo E  (mg/dl) |
| **Intermediate stenosis (n=486)** | |  |  |  |  |  |  |  |  |  |
| Gender | Male | 0.062 | <0.001 | <0.001 | 0.873 | <0.001 | 0.044 | <0.001 | 0.025 | 0.093 |
|  | Female | 0.003 | <0.001 | <0.001 | 0.410 | <0.001 | 0.001 | <0.001 | <0.001 | 0.033 |
| Diabetes | Yes | 0.026 | 0.081 | 0.013 | 0.728 | 0.081 | 0.042 | <0.001 | 0.023 | 0.027 |
|  | No | 0.009 | <0.001 | <0.001 | 0.841 | <0.001 | 0.002 | <0.001 | 0.001 | 0.075 |
| Smoking | Yes | 0.079 | <0.001 | <0.001 | 0.435 | <0.001 | 0.045 | <0.001 | 0.121 | 0.157 |
|  | No | 0.004 | <0.001 | <0.001 | 0.281 | <0.001 | 0.002 | <0.001 | <0.001 | 0.021 |
| Age | ≥65 years | 0.383 | <0.001 | <0.001 | 0.052 | <0.001 | 0.133 | <0.001 | 0.084 | 0.746 |
|  | <65 years | <0.001 | <0.001 | <0.001 | <0.001 | 0.006 | <0.001 | <0.001 | <0.001 | 0.001 |
| **Severe stenosis (n=536)** | |  |  |  |  |  |  |  |  |  |
| Gender | Male | <0.001 | <0.001 | <0.001 | 0.001 | <0.001 | <0.001 | <0.001 | <0.001 | <0.001 |
|  | Female | <0.001 | <0.001 | 0.008 | <0.001 | <0.001 | 0.001 | <0.001 | <0.001 | 0.120 |
| Diabetes | Yes | <0.001 | <0.001 | <0.001 | 0.032 | <0.001 | <0.001 | <0.001 | 0.001 | 0.075 |
|  | No | <0.001 | <0.001 | <0.001 | <0.001 | <0.001 | <0.001 | <0.001 | <0.001 | 0.086 |
| Smoking | Yes | <0.001 | 0.002 | <0.001 | 0.011 | 0.002 | <0.001 | <0.001 | <0.001 | <0.001 |
|  | No | <0.001 | <0.001 | <0.001 | <0.001 | <0.001 | <0.001 | <0.001 | <0.001 | 0.006 |
| Age | ≥65 years | <0.001 | <0.001 | <0.001 | 0.012 | <0.001 | <0.001 | <0.001 | <0.001 | 0.003 |
|  | <65 years | <0.001 | 0.002 | <0.001 | <0.001 | 0.005 | <0.001 | <0.001 | <0.001 | 0.001 |

Table 2. The association of coronary artery stenosis severity with lipoproteins.

|  | Univariate logistic regression | | | Multivariate logistic regression | | |
| --- | --- | --- | --- | --- | --- | --- |
|  | OR | 95%CI | *p* value | OR | 95%CI | *p* value |
| **Model 1 (fasting state)** |  |  |  |  |  |  |
| Age | 1.017 | 1.005-1.030 | 0.007 | 1.019 | 1.005-1.033 | 0.008 |
| Male | 2.144 | 1.659-2.770 | <0.001 | 2.180 | 1.648-2.883 | <0.001 |
| Smoker^1^ | 1.833 | 1.423-2.360 | <0.001 |  |  |  |
| Diabetes mellitus | 2.117 | 1.606-2.790 | <0.001 | 2.002 | 1.482-2.705 | <0.001 |
| Prior history of CHD^2^ | 3.878 | 2.960-5.082 | <0.001 | 3.758 | 2.829-4.993 | <0.001 |
| TC (mmol/L) | 0.833 | 0.743-0.935 | 0.002 |  |  |  |
| LDL-C (mmol/L) | 0.840 | 0.732-0.964 | 0.013 |  |  |  |
| HDL-C (mmol/L) | 0.254 | 0.158-0.406 | <0.001 | 0.262 | 0.158-0.436 | <0.001 |
| **Model 2 (non-fasting state)** |  |  |  |  |  |  |
| Age | 1.017 | 1.005-1.030 | 0.007 | 1.020 | 1.006-1.034 | 0.005 |
| Male | 2.144 | 1.659-2.770 | <0.001 | 2.156 | 1.627-2.857 | <0.001 |
| Smoker^1^ | 1.833 | 1.423-2.360 | <0.001 |  |  |  |
| Diabetes mellitus | 2.117 | 1.606-2.790 | <0.001 | 2.014 | 1.487-2.727 | <0.001 |
| Prior history of CHD^2^ | 3.878 | 2.960-5.082 | <0.001 | 3.809 | 2.860-5.073 | <0.001 |
| TC (mmol/L) | 0.701 | 0.615-0.799 | <0.001 |  |  |  |
| LDL-C (mmol/L) | 0.717 | 0.614-0.837 | <0.001 |  |  |  |
| HDL-C (mmol/L) | 0.186 | 0.113-0.304 | <0.001 | 0.177 | 0.104-0.303 | <0.001 |

Values are expressed as Odds ratios (OR) and 95% confidence intervals (CI). Nagelkerke R Square for multiple logistic regression analysis in model 1 and model 2 were 0.231 and 0.246 respectively. HDL-C, high density lipoprotein cholesterol; LDL-C, low density lipoprotein cholesterol; TC, total cholesterol. ^1^ Smokers include current smokers or former smokers who have quitted cigarette smoking for less than 10 years. ^2^ Prior history of CHD include prior myocardial infarction, prior coronary artery revascularization and documented coronary artery stenosis by angiography.

Table 3. The association of coronary artery stenosis severity with apolipoproteins.

|  | Univariate logistic regression | | | Multivariate logistic regression | | |
| --- | --- | --- | --- | --- | --- | --- |
|  | OR | 95%CI | *p* value | OR | 95%CI | *p* value |
| **Model 3 (fasting state)** |  |  |  |  |  |  |
| Age | 1.017 | 1.005-1.030 | 0.007 | 1.018 | 1.004-1.032 | 0.011 |
| Male | 2.144 | 1.659-2.770 | <0.001 | 2.196 | 1.662-2.902 | <0.001 |
| Smoker^1^ | 1.833 | 1.423-2.360 | <0.001 |  |  |  |
| Diabetes mellitus | 2.117 | 1.606-2.790 | <0.001 | 1.997 | 1.480-2.695 | <0.001 |
| Prior history of CHD^2^ | 3.878 | 2.960-5.082 | <0.001 | 3.824 | 2.880-5.076 | <0.001 |
| ApoA1(g/L) | 0.209 | 0.111-0.395 | <0.001 | 0.198 | 0.099-0.396 | <0.001 |
| ApoB (g/L) | 0.806 | 0.488-1.329 | 0.397 |  |  |  |
| ApoE (mg/dl) | 0.883 | 0.815-0.955 | 0.002 |  |  |  |
| **Model 4 (non-fasting state)** |  |  |  |  |  |  |
| Age | 1.017 | 1.005-1.030 | 0.007 | 1.018 | 1.004-1.033 | 0.010 |
| Male | 2.144 | 1.659-2.770 | <0.001 | 2.117 | 1.597-2.805 | <0.001 |
| Smoker^1^ | 1.833 | 1.423-2.360 | <0.001 |  |  |  |
| Diabetes mellitus | 2.117 | 1.606-2.790 | <0.001 | 2.007 | 1.483-2.717 | <0.001 |
| Prior history of CHD^2^ | 3.878 | 2.960-5.082 | <0.001 | 3.816 | 2.865-5.083 | <0.001 |
| ApoA1(g/L) | 0.143 | 0.075-0.272 | <0.001 | 0.158 | 0.077-0.324 | <0.001 |
| ApoB (g/L) | 0.472 | 0.282-0.821 | <0.001 |  |  |  |
| ApoE (mg/dl) | 0.819 | 0.750-0.895 | <0.001 | 0.880 | 0.801-0.967 | <0.001 |

Values are expressed as Odds ratios (OR) and 95% confidence intervals (CI). Nagelkerke R Square for multiple logistic regression analysis in model 3 and model 4 were 0.222 and 0.244 respectively. Apo, apolipoprotein. ^1^ Smokers include current smokers or former smokers who have quitted cigarette smoking for less than 10 years. ^2^ Prior history of CHD include prior myocardial infarction, prior coronary artery revascularization and documented coronary artery stenosis by angiography

Table 4. The risk for 5-year MACE occurrence and lipoproteins.

|  | Univariate Cox regression | | | Multivariate Cox regression | | |
| --- | --- | --- | --- | --- | --- | --- |
|  | HR | 95%CI | *p* value | HR | 95%CI | *p* value |
| **Model 1 (fasting state)** |  |  |  |  |  |  |
| Coronary artery stenosis severity | 2.284 | 1.604-3.253 | <0 .001 | 1.764 | 1.220-2.551 | 0.003 |
| Age | 1.021 | 1.004-1.038 | 0.013 | 1.022 | 1.006-1.039 | 0.008 |
| Male | 1.589 | 1.125-2.243 | 0.008 |  |  |  |
| Smoker^1^ | 1.611 | 1.172-2.215 | 0.003 | 1.464 | 1.061-2.020 | 0.020 |
| Diabetes mellitus | 2.028 | 1.480-2.779 | <0 .001 | 1.789 | 1.297-2.467 | <0 .001 |
| TG (mmol/L) | 1.093 | 0.961-1.242 | 0.175 |  |  |  |
| TC (mmol/L) | 1.209 | 1.057-1.383 | 0.006 |  |  |  |
| LDL-C (mmol/L) | 1.364 | 1.166-1.596 | <0.001 | 1.592 | 1.349-1.878 | <0.001 |
| HDL-C (mmol/L) | 0.341 | 0.180-0.648 | 0.001 | 0.248 | 0.122-0.504 | <0.001 |
| **Model 2 (non-fasting state)** |  |  |  |  |  |  |
| Coronary artery stenosis severity | 2.284 | 1.604-3.253 | <0 .001 | 1.740 | 1.203-2.517 | 0.003 |
| Age | 1.021 | 1.004-1.038 | 0.013 | 1.021 | 1.004-1.038 | 0.013 |
| Male | 1.589 | 1.125-2.243 | 0.008 |  |  |  |
| Smoker^1^ | 1.611 | 1.172-2.215 | 0.003 | 1.444 | 1.045-1.994 | 0.026 |
| Diabetes mellitus | 2.028 | 1.480-2.779 | <0 .001 | 1.830 | 1.328-2.524 | <0.001 |
| TG (mmol/L) | 1.018 | 0.890-1.165 | 0.790 |  |  |  |
| TC (mmol/L) | 1.102 | 0.946-1.284 | 0.211 |  |  |  |
| LDL-C (mmol/L) | 1.298 | 1.093-1.542 | 0.003 | 1.657 | 1.381-1.987 | <0.001 |
| HDL-C (mmol/L) | 0.191 | 0.095-0.387 | <0.001 | 0.130 | 0.060-0.280 | <0.001 |

Values are expressed as Hazard ratio (HR) and 95% confidence intervals (CI). HDL-C, high density lipoprotein cholesterol; LDL-C, low density lipoprotein cholesterol; TC, total cholesterol; MACE, major adverse cardiovascular events. ^1^Smokers include current smokers or former smokers who have quitted cigarette smoking for less than 10 years.

Table 5. The risk for 5-year MACE occurrence and apolipoproteins.

|  | Univariate Cox regression | | | Multivariate Cox regression | | | |
| --- | --- | --- | --- | --- | --- | --- | --- |
|  | HR | 95%CI | *p* value | HR | 95%CI | *p* value |  |
| **Model 3 (fasting state)** |  |  |  |  |  |  |  |
| Coronary artery stenosis severity | 2.284 | 1.604-3.253 | <0 .001 | 1.772 | 1.226-2.562 | 0.002 |  |
| Age | 1.02 | 1.004-1.038 | 0.013 | 1.025 | 1.008-1.042 | 0.003 |  |
| Male | 1.589 | 1.125-2.243 | 0.008 | 1.448 | 1.021-2.054 | 0.038 |  |
| Smoker^1^ | 1.611 | 1.172-2.215 | 0.003 |  |  |  |  |
| Diabetes mellitus | 2.028 | 1.480-2.779 | <0 .001 | 1.846 | 1.339-2.546 | <0.001 |  |
| ApoA1(g/L) | 0.234 | 0.101-0.541 | 0.001 | 0.199 | 0.083-0.477 | <0.001 |  |
| ApoB (g/L) | 4.246 | 2.413-7.470 | <0.001 | 6.538 | 3.614-11.827 | <0.001 |  |
| ApoE (mg/dl) | 1.013 | 0.925-1.111 | 0.775 |  |  |  |  |
| **Model 4 (non-fasting state)** |  |  |  |  |  |  |  |
| Coronary artery stenosis severity | 2.284 | 1.604-3.253 | <0 .001 | 1.758 | 1.213-2.549 | 0.003 |  |
| Age | 1.021 | 1.004-1.038 | 0.013 | 1.022 | 1.006-1.039 | 0.009 |  |
| Male | 1.589 | 1.125-2.243 | 0.008 |  |  |  |  |
| Smoker^1^ | 1.611 | 1.172-2.215 | 0.003 | 1.384 | 1.003-1.911 | 0.048 |  |
| Diabetes mellitus | 2.028 | 1.480-2.779 | <0 .001 | 1.834 | 1.330-2.529 | <0.001 |  |
| ApoA1(g/L) | 0.107 | 0.044-0.258 | <0.001 | 0.128 | 0.054-0.305 | <0.001 |  |
| ApoB (g/L) | 3.689 | 1.961-6.943 | <0.001 | 5.350 | 2.793-10.249 | <0.001 |  |
| ApoE (mg/dl) | 0.967 | 0.877-1.066 | 0.498 |  |  |  |  |

Values are expressed as Hazard ratio (HR) and 95% confidence intervals (CI). Apo, apolipoprotein. MACE, major adverse cardiovascular events. ^1^Smokers include current smokers or former smokers who have quitted cigarette smoking for less than 10 years.

Table 6. Demographic and clinical characteristics of Chinese CHD patients with intermediate or severe stenosis according to the tertiles of non-fasting HDL-C level.

|  | 1^st^ tertile^1^(n=353) | 2^nd^ tertile^2^(n=335) | 3^rd^ tertile^3^(n=334) | *p* value^4^ |
| --- | --- | --- | --- | --- |
| Age (years) | 66 [60, 72] | 68[61, 75] | 68[62, 76] | 0.046 |
| Male (gender) | 230(65.16%) | 208(62.09%) | 188(56.29%) | 0.054 |
| BMI (kg/m^2^) | 25.26[23.25, 27.10] | 24.80[22.59, 26.81] | 23.68[21.69, 25.71] | <0.001 |
| **Severity of stenosis** |  |  |  |  |
| Intermediate stenosis | 116(32.86%) | 170(50.75%) | 200(59.88%) | <0.001 |
| Severe stenosis | 237(67.14%) | 165(49.25%) | 134(40.12%) | <0.001 |
| **Medical history** |  |  |  |  |
| Smoker^5^ | 160(45.33%) | 134(40.00%) | 131(39.22%) | 0.208 |
| Hypertension | 248(70.25) | 227(67.76%) | 229(68.56%) | 0.770 |
| Diabetes mellitus | 109(30.88%) | 104(31.04%) | 97(29.04%) | 0.822 |
| Prior history of CHD^6^ | 159(45.04%) | 123(36.72%) | 122(36.53%) | 0.036 |
| History of atrial fibrillation | 47(13.31%) | 42(12.54%) | 44(13.17%) | 0.950 |
| COPD | 26(7.37%) | 22(6.57%) | 24(7.19%) | 0.913 |
| Family history of CVD | 185(52.41%) | 178(53.13%) | 174(52.10%) | 0.963 |
| **Medications** |  |  |  |  |
| Aspirin and/or thienopyridine | 340(96.32%) | 321(95.82%) | 313(93.71%) | 0.235 |
| Anticoagulants | 46(13.03%) | 42(12.54%) | 44(13.17%) | 0.967 |
| Beta blocker | 89(25.21%) | 79(23.58%) | 80(23.95%) | 0.871 |
| ACEI/ARB | 98(27.76%) | 90(26.87%) | 94(28.14%) | 0.930 |
| CCB | 41(11.61%) | 48(14.32%) | 44(13.17%) | 0.569 |
| Statins | 292(82.72%) | 269(80.30%) | 241(72.16%) | 0.002 |
| Other lipid-lowering drugs^7^ | 22(6.23%) | 25(7.46%) | 26(7.78%) | 0.705 |
| **Fasting lipids** |  |  |  |  |
| TC (mmol/L) | 3.74 [3.14, 4.355] | 4.05[3.46, 4.70] | 4.40[3.72, 5.23] | <0.001 |
| TG (mmol/L) | 1.75 [1.27, 2.39] | 1.46[1.10, 2.01] | 1.27[0.99, 1.82] | <0.001 |
| LDL-C (mmol/L) | 2.04[1.52, 2.67] | 2.30[1.82, 2.84] | 2.50[1.90, 3.13] | <0.001 |
| HDL-C (mmol/L) | 0.77[0.69, 0.87] | 0.98 [0.89, 1.06] | 1.27[1.12, 1.40] | <0.001 |
| **Laboratory variables** |  |  |  |  |
| Troponin I (ng/ml) | 0.01[0.00, 0.05] | 0.00 [0.00, 0.01] | 0.00 [0.00, 0.01] | <0.001 |
| BNP (pg/ml) | 63.00[31.00, 150.00] | 55.00[26.00, 143.00] | 54.50[27.00, 135.25] | 0.344 |
| CRP (mg/L) | 1.66[1.28, 5.49] | 1.31[1.28, 4.61] | 1.55[1.28, 5.71] | 0.243 |
| Creatine level (μmol/L) | 83.00[68.00, 97.00] | 80.00[68.00, 96.00] | 75.00[65.00, 90.00] | <0.001 |

Values are expressed as percentage or median [first quartile, third quartile]. ^1^ 1^st^ tertile of non-fasting HDL-C is <0.86mmol/L; ^2^ 2^nd^ tertile of non-fasting HDL-C is 0.86-1.07 mmol/L; ^3^ 3^rd^ tertile of non-fasting HDL-C is >1.07 mmol/L; ^4^ *p* value for differences among three group; ^5^ Smokers include current smokers or former smokers who have quitted cigarette smoking for less than 10 years. ^6^ Prior history of CHD include prior myocardial infarction, prior coronary artery revascularization and documented coronary artery stenosis by angiography. ^7^ Other lipid-lowering drugs include cholesterol absorption inhibitor, fibrates, fish oil ect.

**Figure legends**

**Fig. 1.** Correlations between HDL-C, LDL-C, non–HDL-C, Apo A1, and Apo B in either fasting or non-fating state. They were assessed by Spearman’s correlation coefficient.

**Fig. 2.** Receiver Operating Characteristic (ROC) for evaluation of non-HDL-C associated with MACE free survival in Chinese CHD patients. The cutoff value of non-fasting HDL-C associated with 5-year MACE free survival was >0.942 mmol/L with 54.8% sensitivity and 66.7% specificity [Area Under Curve (AUC): 0.627, CI 95% 0.579-0.674, p<0.01].



 **Fig. 1.**


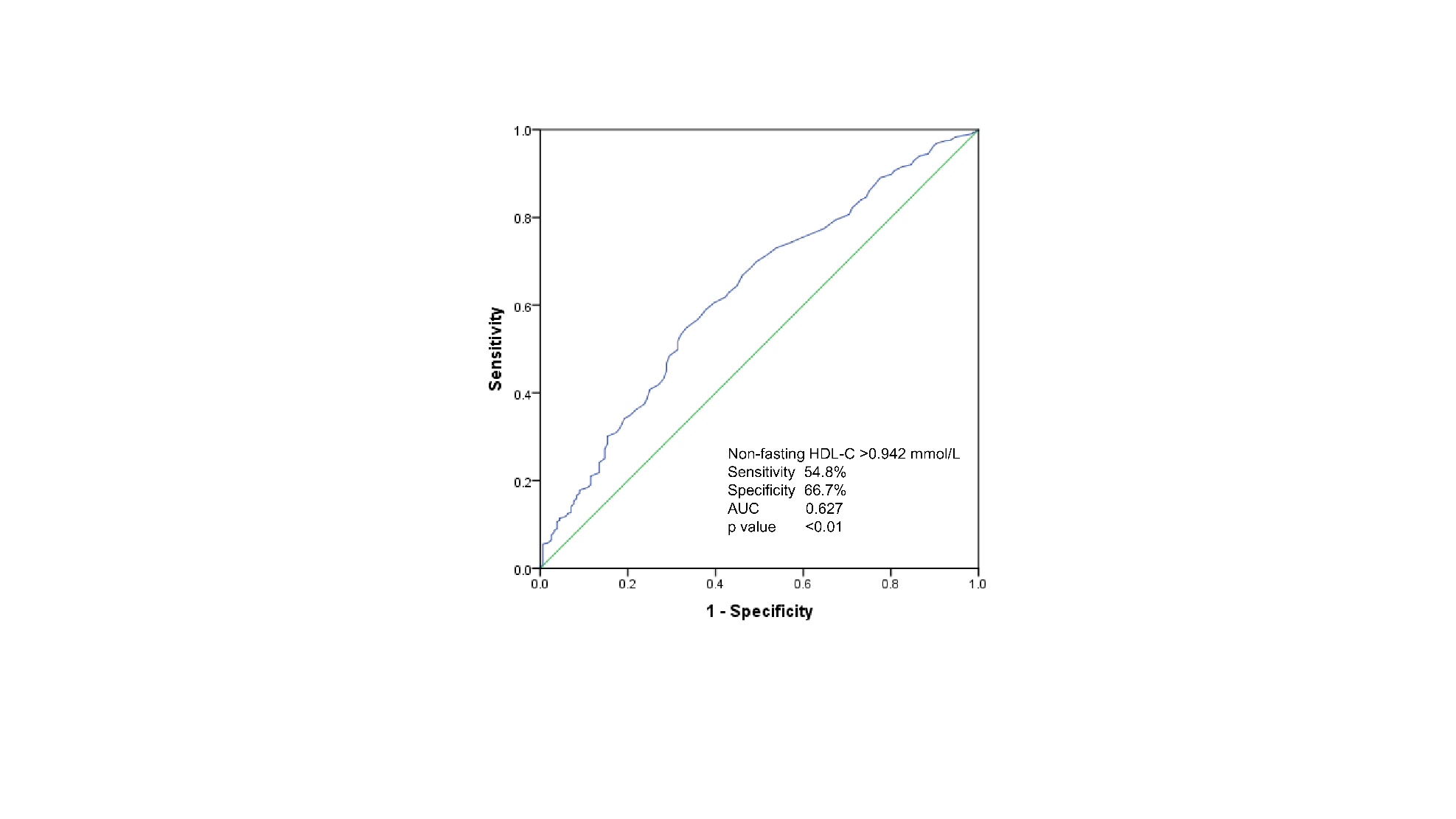
**Fig. 2.**
